# The p53 Modulated Cytotoxicity of *Ophiocoma scolopendrina* Polysaccharide Against Resistance Ovarian Cancer Cells

**Published:** 2019

**Authors:** Elaheh Amini, Javad Baharara, Mahbube Afzali, Najme Nikdel

**Affiliations:** 1. Department of Cellular & Molecular Biology, Faculty of Biological Sciences, Kharazmi University, Tehran, Iran; 2. Department of Biology, Research Center for Animal Development Applied Biology, Mashhad Branch, Islamic Azad University, Mashhad, Iran; 3. Department of Biology, Mashhad Branch, Islamic Azad University, Mashhad, Iran

**Keywords:** Apoptosis, Ovarian neoplasms, Paclitaxel

## Abstract

**Background:**

Marine environment is a valuable source of bioactive compounds with variable medicinal properties. Previously, it was shown that *Ophiocoma erinaceus* extracted polysaccharide has prominent cytotoxic effect on HeLa human cervical cancer cells. In the present study, the anti-cancer properties of polysaccharide extracted from *Ophiocoma scolopendrina* (*O. scolopendrina)* were examined in comparison with paclitaxel as a conventional drug against resistant ovarian cancer; also, its related mechanism against A2780cp ovarian cancer cells was investigated.

**Methods:**

The A2780cp cancer cells and NIH3T3 normal cells were cultured and treated with different concentrations of polysaccharide extracted from *O. scolopendrina* for 24 *hr* and 48 *hr*. Then, cell toxicity was studied by MTT assay, morphology of cells was observed under inverted microscopy and the type of induced cancer cell death was assessed by annexin V-FITC, propodium iodide and acridine orange staining. Finally, the apoptosis pathway was determined by measurement of caspase-3 and caspase-9 activity and assessment of p53 and Bcl-2. The statistical analysis was performed by SPSS software, one way ANOVA and p<0.05 was considered significant.

**Results:**

Our observations from MTT assay and morphological assessment exhibited that *O. scolopendrina* isolated polysaccharide inhibited proliferation of ovarian cancer cells with IC_50_ of 35 *μg/ml*, while paclitaxel suppressed tumor cell growth with IC_50_=10 *μg/ml*. In contrast, MTT observations revealed low cytotoxicity of these chemotherapeutic agents against NIH3T3 normal cells. Also, the analysis correlated with induced cell death elucidated that concurrent treatment of polysaccharide plus paclitaxel had a further anti-cancer effect against A2780cp cells mainly through restoration of p53 and mitochondrial apoptosis cell death induction.

**Conclusion:**

Taken together, our research supports the finding that application of polysaccharide extracted from *O. scolopendrina* can be considered a promising marine chemotherapeutic approach for advancing efficacy of paclitaxel in treatment of resistant ovarian cancer. Additional *in vivo* experiments are required to elucidate the role of brittle star polysaccharides in animal and clinical trials.

## Introduction

Cancer is a chronic and fatal disease worldwide and ovarian cancer is the fourth women deadly disorder, especially in older women 1,2. Current therapeutic ovarian cancer methods include surgery and the use of chemotherapy is limited due to harmful side effects 3,4. Chemotherapeutic drugs have created multidrug resistance that induces noticeable challenge for oncologists in treatment of cancer 5. To date, several chemotherapeutic drugs have been introduced to interfere with ovarian carcinoma, but among suggested anti-cancer drugs, paclitaxel is a favorable anti-tumor agent which creates cytotoxicity *via* apoptosis triggering in many tumors, particularly in ovarian cancer 6. Nevertheless, there are unwanted complexities for ovarian cancer treatment 3. Thus, many researches attempted to find novel therapeutic methods to overcome ovarian drug resistance 4,5.

Natural products occupy the main source of chemotherapeutic agents in the past few decades that have attracted researchers’ attentions 7. The low toxicity and minimum levels of side effects in natural products give them priority in comparison to synthetic chemotherapeutic leads 8. Among various biological effects of natural metabolites, their capacity for inhibition of cancer can be noted as the therapeutic importance 7.

Polysaccharides are biocompounds that have antioxidant, anti-microbial and anti-coagulant activities 9. In addition, in recent years, a growing body of investigations concentrated on anti-cancer potential of natural polysaccharides 10. The used mechanisms by these compounds are tumor growth suppression, apoptosis induction and metastasis prevention 11. Apoptosis or programmed cell death is a process that is commonly found in living organisms for eradication of redundant or damaged cells 12. The morphological features in apoptotic cells are shrinkage of cell membrane, DNA fragmentation and apoptotic body generation 13.

The ocean is composed of tremendous structurally bioactive substances with various pharmaceutical properties 14. Marine flora and fauna are unique sources of natural compounds with biological properties such as anti-microbial, anti-viral, antioxidant and anti-cancer effects 15. Aquatic echinoderms are accounted as abundant marine invertebrates which contain physiologically active metabolites required in biomedicine 16.

Among biomedical investigation in echinoderms (composed of Asteroidea: sea stars or starfish, Crinoid: crinoids, Ophiuroidea: brittle stars, Echinoidea: sea urchins and Holothuroidea: sea cucumbers), brittle stars (Ophiuroidea) possess unknown bioactive substances rather than starfish (Asteroidea) and sea cucumbers (Holothuroidea) that are related to their therapeutic properties and fewer studies have been conducted on such properties in the past three decades 14. Brittle star (Ophiuroidea) is an aquatic invertebrate with the capacity for arm regeneration 17. To date, the presence of some bioactive substances such as terpenes, sulfated sterols, carotenoid sulfate, phenylpropanoids and naphthoquinones in brittle star have been proved which may be important in anti-cancer therapy 18.

Considering the bioactivity of marine compounds, this study was designed to investigate the concomitant cytotoxic effect of polysaccharide isolated from Persian Gulf brittle star *Ophiocoma scolopendrina (O. scolopendrina)* and paclitaxel on A2780cp cells and their related mechanism against human ovarian cancer cells.

## Materials and Methods

### Reagents

A2780cp cell line (Human epithelial ovarian carcinoma) was purchased from NCBI (National Cell Bank of Iran). Trypsin/EDTA (1X) and fetal bovine serum were provided from Gibco (USA), RPMI-1640 and Trypan blue were purchased from Bio idea (Iran), and penicillin/streptomycin and phosphate buffer saline were purchased from PAA (Austria). PI (Propodium Iodide), DAPI (4′, 6-diamidino-2-phenylindole dihydrochloride) kit and MTT [3-(4, 5-dimethylthiozol-2-il) 2, 5 di phenyl tetrazolium bromide] and acridine orange/propodium iodide were purchased from Sigma (USA). Annexin V-FITC kit and Caspase-9 assay and Caspase-3 assay kit were prepared from Abcam (UK). Taxol or paclitaxel was purchased from Sigma (USA).

### Extraction of polysaccharide

Firstly, morphometric estimation of *O. scolopendrina* was conducted at the Research Center of Applied Biology at Mashhad Branch of Islamic Azad University. Then, specimens were washed and dried in the dark. Then, 500 *gr* dried brittle star were added to 100 *ml* water, boiled for 3 *hr* and centrifuged and filtered. In the next step, 3 volumes of 95% (*v/v*) ethanol were added and incubated at 4*°C* overnight. Then, specimen was centrifuged and the precipitate was dissolved in distilled water and centrifuged for 20 *min* to get aqueous supernatant lyophilized (total polysaccharide) 19.

### Cell culture

The A2780cp cells and NIH3T3 fibroblast normal cells were cultured in RPMI 1640, DMEM medium with 10% FBS and 1% penicillin/streptomycin in incubator at 37*°C* containing 5% CO_2_, respectively.

### MTT assay

A2780cp cells and NIH3T3 were cultured and treated with different concentrations of extracted polysaccharide from *O. scolopendrina* (12.5, 25, 50 *μg/ml*), taxol (5, 10, 20, 40 *μg/ml*) and synergism treatment was done by brittle star polysaccharide and taxol (12.5 *μg/ml* polysaccharide +10 *μg/ml* taxol, 12.5 *μg/ml* polysaccharide +25 *μg/ml* taxol) for 24 and 48 *hr*. After a certain time, the cells were incubated with MTT for 4 *hr* and DMSO was added to dissolve formazan according to the company’s protocol. Finally, the absorbance of each well was measured at a wavelength of 560 *nm* by a spectrophotometer.

### Evaluation of induced cell death

***DAPI staining:*** 4′, 6-diamidino-2-phenylindole dihydrochloride staining was used to evaluate the morphology of nuclei. In this assay, A2780cp ovarian cancer cells were seeded on a coverslip and incubated with the desired concentration of brittle star polysaccharide and paclitaxel (IC_50_) for 24 *hr*. Then, the cells were washed with PBS and DAPI was added and cells were incubated for 10 *min* in the dark and morphological changes were observed under the fluorescence microscope.

### Acridine orange/propodium iodide staining

The apoptotic morphological characteristics of cells were ascertained by acridine orange/propodium iodide staining. The ovarian tumor cells were cultured and treated with IC_50_ concentration of isolated polysaccharide and taxol and synergism treatment for 24 *hr*. Then, the cells were washed with PBS and acridine orange (20 *μg/ml*) and propodium iodide (20 *μg/ml*) were added (1:1). Eventually, the cells were observed under a fluorescence microscope.

### Annexin V-FITC-PI

Annexin/PI method was used to assess phosphatidylserine exclusion on the extracellular side of apoptotic cells. For this purpose, the A2780cp cells were seeded and treated with IC_50_ concentrations of polysaccharide and paclitaxel, according to the company’s protocol. Subsequently, the tumor cells were suspended in 500 *μl* 1X binding buffer. Thereafter, 5 *μl* annexin V-FITC and 5 *μl* propodium iodide were added and incubated for 5 *min* at room temperature in the dark. Then, they were analyzed using flow cytometry.

### PI staining

The evaluation of apoptosis was performed using PI assay. The A2780cp cells were cultured and treated with IC_50_ concentrations of polysaccharide alone or in synergism state for 24 *hr* and cell suspension was centrifuged and mixed with 700 *μl* PI solution for 20 *min* in the dark. Next, the fluorescence of stained cells was evaluated using a FACScan laser flow cytometer.

### Caspase-3 and Caspase-9 assay

Ovarian cells were cultured and treated with anti-proliferative concentrations of isolated polysaccharide or paclitaxel for 24 *hr*. Then, cell lysis buffer, 2X reaction buffer and LEHD-pNA substrate were added to untreated and treated cells according to the kit protocol. At last, the absorbance was read at 405 *nm* using plate reader.

### Apoptosis related mRNA expression

The expression of p53 and Bcl-2 mRNA was analyzed by RT-PCR. The total cellular RNAs of ovarian cancer cells treated with or without extracted polysaccharide, paclitaxel and combination treatment were isolated using the high pure RNA isolation kit (Roche, Germany) and reverse transcribed to cDNA using the easy cDNA synthesis kit (Pars Tous, Iran) according to manufactures protocol. Then, RT-PCR was performed with 10× buffer, MgCl_2_ 25 *mM*, d NTP, and Taq DNA polymerase. The forward and reverse primer sequence was: 5′ CCAGGGCAGCTACGGTTTC 3′ forward for p53 and 5′ CTCCGTCATGTGCTGTGACTG 3′ reverse for p53. The forward and reverse primer sequences for Bcl-2 were designed as 5′ CATGTGTGTG GAGAGCGTCAAC 3′ and 5′ CAGATAGGCACCCAGGGTGAT 3′. Following amplification, PCR products were subjected to electrophoresis in a 2% agarose gel.

### Data analysis

The statistical analysis was performed by SPSS software, one way ANOVA and p<0.05 was considered significant.

## Results

### MTT assay

**Figure 1.**
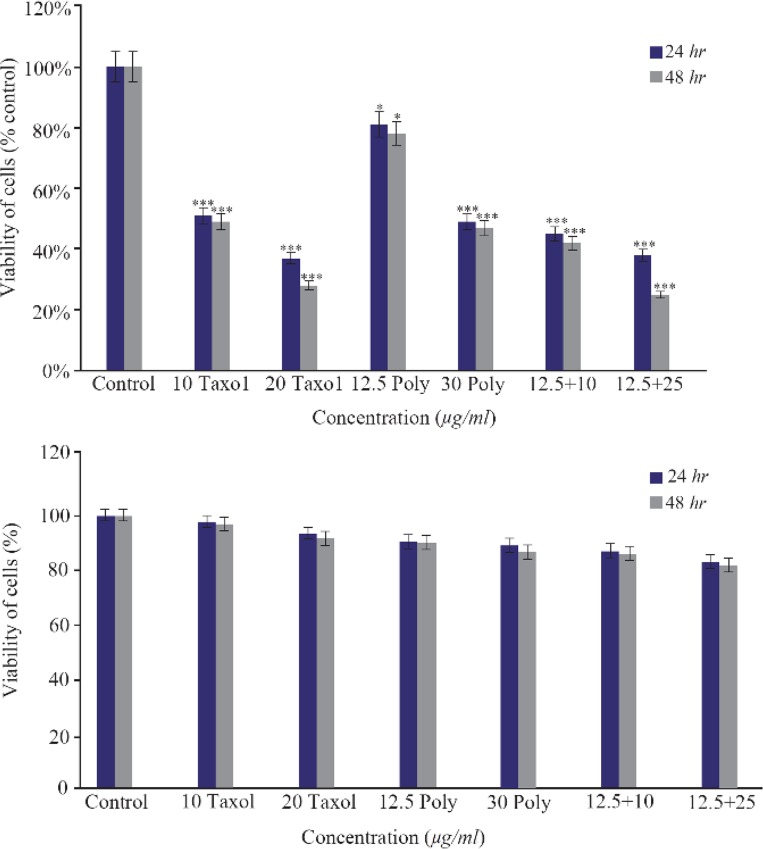
The cytotoxic effect of brittle star polysaccharide and paclitaxel on A2780cp cancer cells (Upper) and NIH3T3 normal cells (Lower) were studied with MTT assay. The cancer and normal cells were classified in two parts, control (Culture medium RPMI 10%) and treatment groups which were exposed to different concentrations of brittle star polysaccharide and paclitaxel for 24 and 48 *hr*. The data are represented as mean±SD and *p<0.05, **p<0.005 and ***p<0.001 were considered significant.

MTT analysis showed that treatment with brittle star polysaccharide at concentration of 30 *μg/ml* (p<0.01) could be considered as IC_50_. Incubation with various concentrations of taxol exhibited that paclitaxel inhibited 50% of ovarian cancer cell growth at 10 *μg/ml* (p<0.01). However, treatment with extracted polysaccharide and paclitaxel simultaneously exerted more cytotoxicity on ovarian cancer cells, so that IC_50_ concentration was determined as 12.5 *μg/ml* and 10 *μg/ml* (p<0.01) for polysaccharide and taxol, respectively (Figure 1). Further, there was no significant cytotoxicity of brittle star polysaccharide and taxol on NIH3T3 normal cells.

### Morphological observations by inverted microscope

**Figure 2.**
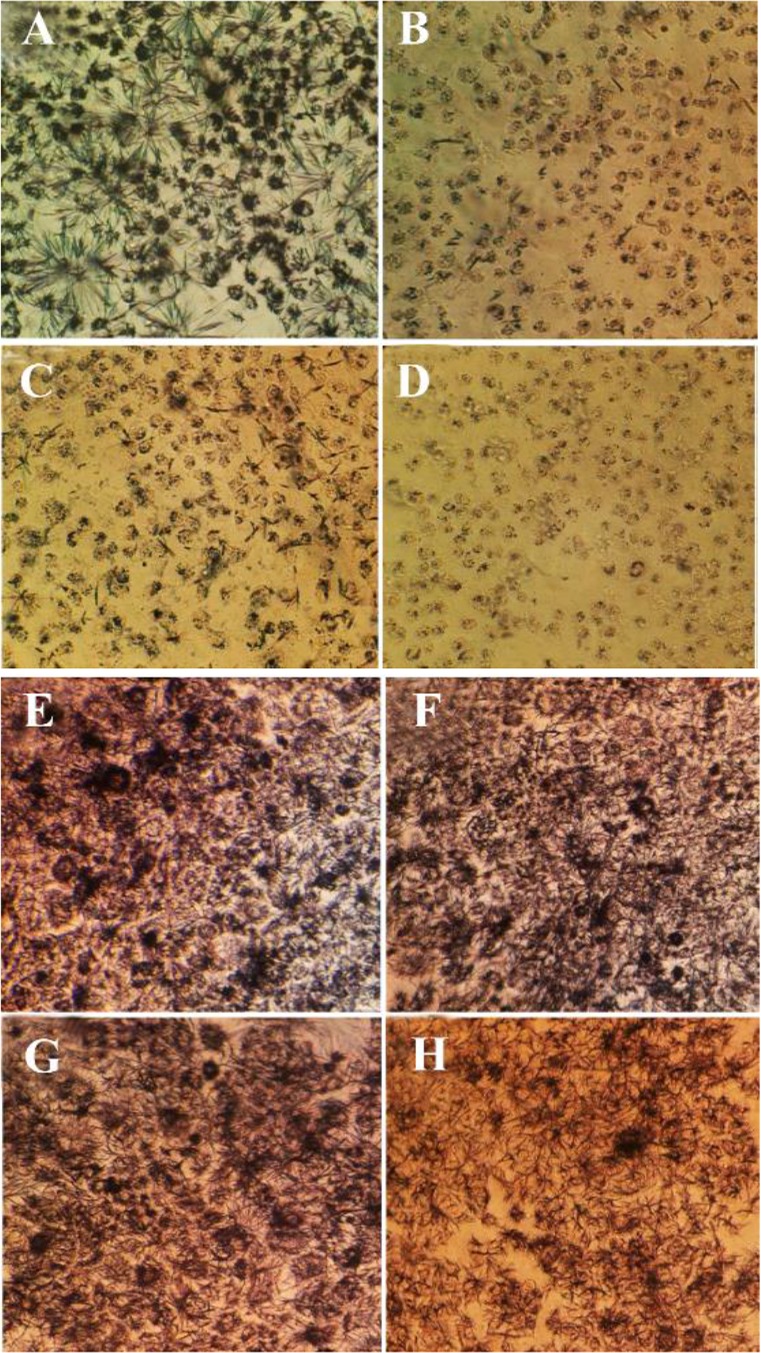
Morphological alterations of A2780cp ovarian cancer cells and NIH3T3 fibroblast cells under exposure to inhibitory concentrations of *O. scolopendrina* polysaccharide and taxol using MTT assay. Reduction of the amount of formazan crystals in cancer cells compared with the untreated cells demonstrated anti-proliferative effect under inverted microscope (Magnification ×200). The emergence of high formazan crystals in NIH3T3 treated cells revealed the effect of extracted polysaccharide and taxol on ovarian cancer cells. A, E) Control, B, F) 10 *μg/ml* taxol, C, G) 30 *μg/ml* polysaccharide, D, H) 10 *μg/ml* taxol +12.5 *μg/ml* polysaccharide.

As exhibited in figure 2, the anti-proliferative activity of brittle star polysaccharide and taxol in IC_50_ concentration on A2780cp cells induced apparent morphological alterations such as reduction of cell volume, cell shrinkage and apoptotic body formation which induced apoptosis in exposure with cytotoxic dosage of brittle star polysaccharide and paclitaxel. Meanwhile, NIH3T3 treated cells didn’t show considerable alterations. High quantity of formazan crystals validated the effect of brittle star polysaccharide and taxol on ovarian cancer cells.

### DAPI staining

**Figure 3.**
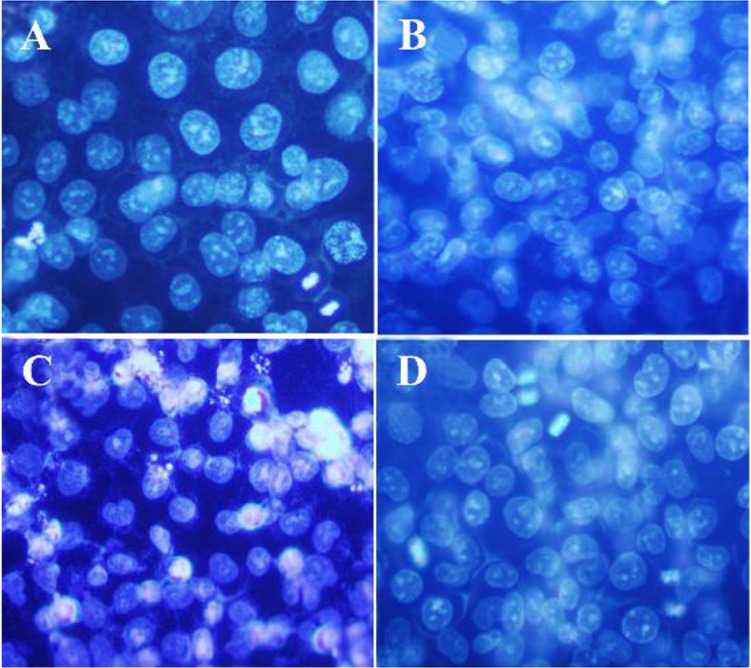
DAPI staining, changes in cell nucleus indicating nuclear fragmentation as clear features of apoptosis in treated cells with 50% inhibitory concentrations of polysaccharide and taxol, under fluorescence microscopy (Magnification ×400). A) Control, B) 10 *μg/ml* taxol, C) 30 *μg/ml* polysaccharide, D) 10 *μg/ml* taxol +12.5 *μg/ml* polysaccharide.

DAPI staining was used to study the nucleus morphological changes under treatment with isolated polysaccharide and paclitaxel. As shown in figure 3, the A2780cp treated cells (By IC_50_ concentration of brittle stars polysaccharide, paclitaxel and synergism treatment) revealed DNA fragmentation which confirmed pro-apoptotic effect of brittle stars polysaccharide and paclitaxel, alone or in combination (Figure 3).

### Acridine orange/propodium iodide staining

**Figure 4.**
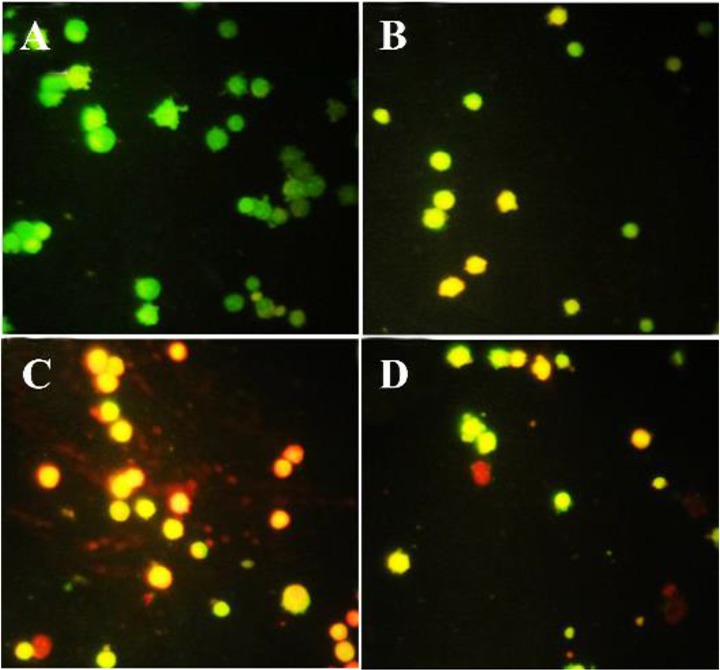
Acridine orange/propodium iodide staining indicated the apoptosis induction under treatment with polysaccharide and taxol. The untreated cells are green and indicator of live cells, yellow and orange color indicates apoptosis (Magnification ×400). A) Control, B) 10 *μg/ml* taxol, C) 30 *μg/ml* polysaccharide, D) 10 *μg/ml* taxol +12.5 polysaccharide *μg/ml*.

After treatment, acridine orange/propodium iodide staining was conducted to distinguish apoptosis or necrosis induction under exposure with brittle star polysaccharide and paclitaxel. In this assay, green color is a crucial mark of live cells and red color is indicator of dead or necrotic cells. As shown in figure 4, control group showed green cells; meanwhile in treated cells with IC_50_ concentrations of brittle stars polysaccharide, taxol and simultaneous treatment, more cells were apoptotic, which confirmed involvement of apoptosis in cytotoxicity of brittle stars polysaccharide and taxol and their effect on A2780cp cancer cells (Figure 4).

### Annexin V-FITC/PI

**Figure 5.**
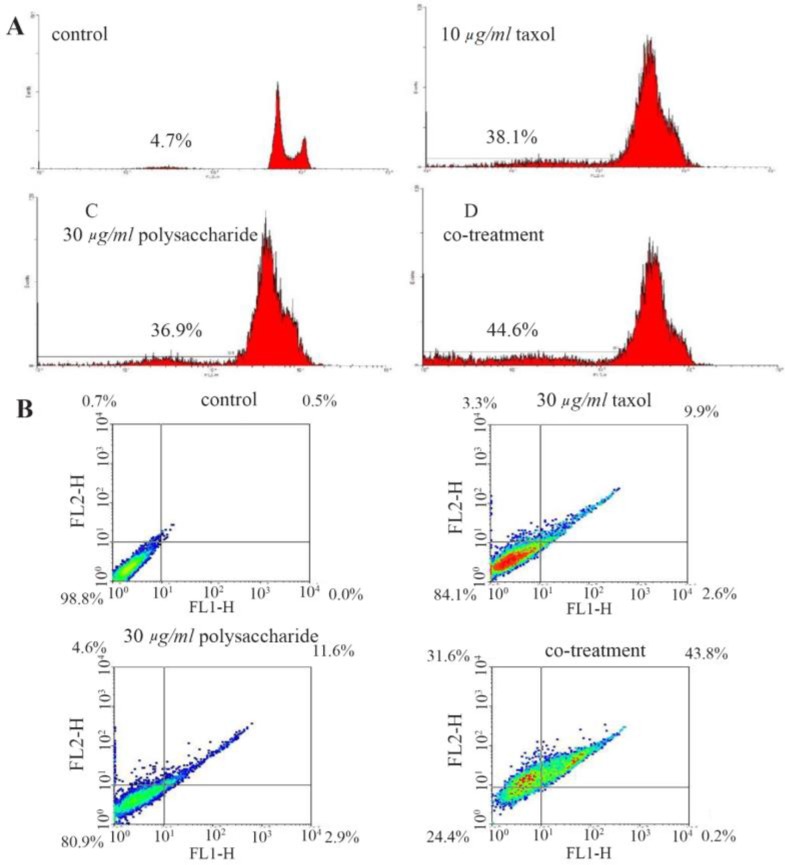
A) Histograms obtained by the annexin V-FITC / PI kit indicating apoptosis induction in A2780cpcells treated with *O. scolopendrina* polysaccharide and paclitaxel. B) PI assay demonstrated sub-G1 peak that is one of most important indicators of apoptosis induced by polysaccharide and paclitaxel. Co-treatment: 10 *μg/ml* taxol +12.5 *μg/ml* polysaccharide.

According to results obtained by flow cytometric analysis of annexin V-FITC/PI kit, the frequent portion of cell death induced by IC_50_ concentrations of brittle stars polysaccharide and paclitaxel alone or in combination was apoptosis which is indicated in figure 5A.

### PI assay

In PI assay, apoptotic cells showed a sub-G1 peak. Results from this assay showed that brittle stars polysaccharide and taxol in IC_50_ dosage evoked apoptosis in A2780cp ovarian tumor cells (Figure 5B).

### Caspase-3 and caspase-9 colorimetric assay

**Figure 6.**
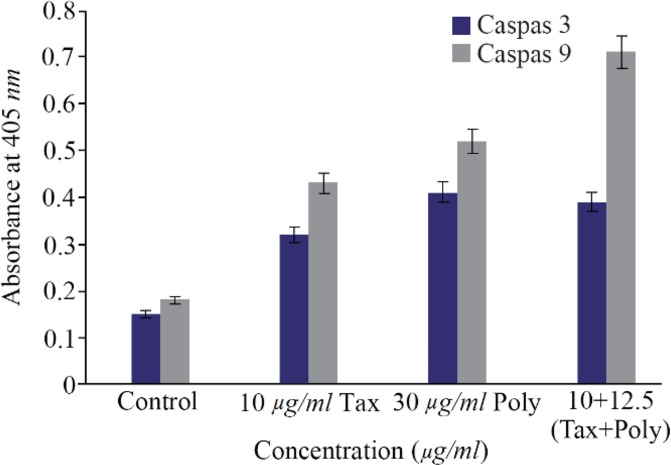
The measurement of caspase-3 and -9 activity in A2780cp cells treated with *O. scolopendrina* polysaccharide and paclitaxel. To determine whether apoptosis induction is caspase-dependent, caspas-3 and caspase-9 assay was used. Histogram represented that the caspase-3 and -9 activity increased under incubation with brittle star polysaccharide and paclitaxel as compared to untreated cells. As shown, the increment of caspase-3 and caspase-9 activity revealed that the *O. scolopendrina* polysaccharide can improve the cytotoxic effect of taxol *via* intrinsic apoptosis pathway.

Caspase-3 and caspase-9 colorimetric assays were conducted to determine the direction of apoptosis induced by polysaccharide and paclitaxel. The results showed that the induced apoptosis pathway by desired dosage of brittle stars polysaccharide, taxol and concurrent treatment of them was caspase-dependent indicating the intrinsic pathway induced by brittle star polysaccharide and paclitaxel (Figure 6).

### p53 and Bcl-2 expression

**Figure 7.**
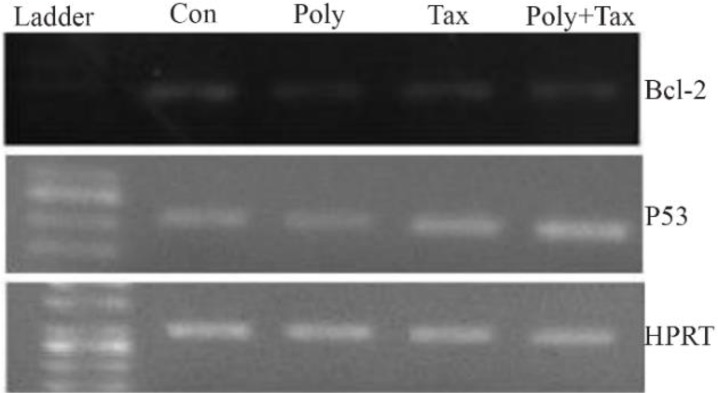
A2780cp cells were treated with *O. erinaceus* extracted polysaccharide, taxol and synergistic treatment. The mRNA expressions of Bax and Bcl-2 were assessed by RT-PCR that demonstrated cytotoxic effect of polysaccharide, taxol and co treatment via intrinsic pathway (con= control or untreated cells, poly=30 *μg/ml* brittle star polysaccharide, tax=10 *μg/ml* taxol or paclitaxel, poly+tax=co treatment of 10 *μg/ml* taxol and 12.5 *μg/ml* polysaccharide).

The analysis of transcriptional levels of two apoptotic-related genes, p53 and Bcl-2 in human ovarian cancer cells incubated with IC_50_ concentrations of brittle star polysaccharide, paclitaxel and co treatment showed that the pretreatment of A2780cp cells with polysaccharide, taxol and synergistic treatment down regulated Bcl-2 expression and up regulated p53 mRNA level (Figure 7).

## Discussion

In our study, the cytotoxic effect and the type of cell death induced by polysaccharide extracted from brittle star *O. scolopendrina* were investigated and its concurrent effect with paclitaxel against ovarian cancer cells was evaluated. A2780cp cell line is a human epithelial ovarian carcinoma and taxol is used as a usual, approved and appreciable chemotherapeutic drug for treatment of some cancers like ovarian cancer. This agent was used in this study (As positive control) to assess anti-cancer efficacy of the brittle star polysaccharide against ovarian cancer cells.

So, the single and synergistic effect of brittle star polysaccharide and paclitaxel on A2780cp human ovarian cancer cells was examined. The morphological observation and MTT assay exhibited that the derived polysaccharide induced an anti-growth effect (IC_50_=30 *μg/ml*) in a dose-time dependent manner on A2780cp cells and taxol exerted 50% cytotoxicity in concentration of 10 *μg/ml*. The PI, annexin V-FITC, DAPI and acridine orange/propodium iodide assay showed that the combination treatment of *O. scolopendrina* polysaccharide and taxol induced more apoptosis. Besides, the conducted experiments using caspase-3 and caspase-9 enzymatic activity showed that the apoptosis induced by brittle star extracted polysaccharide and paclitaxel in A2780cp cells was caspase-dependent or related to mitochondrial pathway. As activation of p53 stimulates apoptosis, up-regulation of p53 and down regulation of Bcl-2 under treatment with *O. scolopendrina* polysaccharide and taxol may be an effective mechanism of cell death in resistance of A2780 cells.

Results obtained in other studies related to the biological characteristics of the natural polysaccharides, confirmed the anti-cancer effect of these compounds. Chen *et al* in 2013 documented medicinal properties of polysaccharide extracted from fungus *Rhizopus nigricans* and revealed that this isolated polysaccharide inhibited human gastric cancer cell growth -BGC-823 using apoptosis increment *via* mitochondrial mediated pathway 20.

Lavi *et al* in 2006 reported anti-tumor effect of polysaccharide fraction from mushroom *Pleurotus ostreatus* on HT-29 cell line 21. Cao *et al* exhibited that *Angelica sinensis* polysaccharide induced apoptosis *via* intrinsic pathway in HeLa cervical cancer cells *in vitro* and *in vivo* 22.

In addition, there are evidences on the basis of utilization of natural polysaccharides from flora of terrestrial ecosystem in biomedicine 10,11,23. Gamaleldeen in 2009 evaluated the biological effect of various fractions of polysaccharide from brown algae *Sargassum latifolium* and proved that E3 fraction indicated anti-cancer activity against leukemia cells as compared with E1, E2 and E4 fraction 24. Lee *et al* in 2011 examined the anti-metastatic potential of polysaccharide extracted from *Asterina pectinifera* in MDA-MB-231 breast cancer cells and displayed that extracted polysaccharide had tumor growth inhibitory effect on examined breast cancer cells 25.

Lu *et al* in 2012 demonstrated the tumor inhibitory effect of *Coix lacrymajobi* (Adlay seed) polysaccharide fraction against A549 cancer cells and reported that intrinsic apoptosis pathway was responsible for this cytotoxicity 26. In 2014, Wang *et al* reported that *Boschniakia rossica* polysaccharide suppressed Hep2 cell line proliferation with G0/G1 cell cycle arrest and elicited reprogrammed cell death through mitochondrial pathway 27. Furthermore, Thangam *et al* displayed the growth inhibitory effect of polysaccharide fractions extracted from *Cymbopogon citratus* against LN-cap and Siha tumor cells 28.

p53 is a tumor suppressor and a nuclear transcription factor which exhibits loss of function mutations in part of cancer cell types. Existence of mutation in p53 conferred chemo-resistance phenotype in malignant cells. Oncological studies showed that accumulation of p53 in tumor cell nucleus elicits pro-apoptotic activity 29. Yazdanpanahi *et al* elucidated that increase in p53 gene expression is a molecular mechanism responsible for natural compound cytotoxicity in breast cancer cells 30. In a previous study, it was indicated that *Ophiocoma erinaceus* (*O. erinaceus*) polysaccharide can be an appropriate cytotoxic compound against human cervical cancer cells 19. Consequently, all experiments performed in this field in agreement with our observations confirmed the importance of natural polysaccharides in the treatment of lethal diseases such as cancer.

## Conclusion

In this study, the anti-cancer effect of extracted poly saccharide from brittle star *O. scolopendrina* and taxol (chemotherapeutic drug) alone and in combination was evaluated against resistant ovarian cancer *in vitro*. According to our observations, polysaccharide extracted from *O. scolopendrina* and paclitaxel induced apoptosis cell death *via* intrinsic caspase-dependent pathway in ovarian cancer cells. It can be suggested that this marine polysaccharide was a worthy anti-cancer candidate to increase the anti-tumor efficacy of paclitaxel for treating human ovarian cancer, but additional investigations are needed to assess clinical oncological studies.
